# Colorectal endoscopic submucosal dissection in the USA: The current state and future perspectives

**DOI:** 10.1002/deo2.394

**Published:** 2024-06-23

**Authors:** Abdulrahman Qatomah, Hiroyuki Aihara

**Affiliations:** ^1^ Division of Gastroenterology and Hepatology McGill University Health Center Montreal Canada; ^2^ Division of Gastroenterology and Hepatology King Faisal Specialist Hospital and Research Center Jeddah Saudi Arabia; ^3^ Division of Gastroenterology, Hepatology and Endoscopy Brigham and Women's Hospital, Harvard Medical School Boston Massachusetts USA

**Keywords:** education, endoscopic submucosal dissection, robotic endoscopy, traction, training

## Abstract

Endoscopic submucosal dissection (ESD) is a transformative advancement in the endoscopic management of superficial gastrointestinal lesions. Initially conceived for the treatment of early gastric cancer, ESD has demonstrated proficiency in achieving en‐bloc resection of superficial gastrointestinal lesions. ESD has experienced widespread acceptance in Japan and East Asia; however, its adoption in the USA remains delayed. This initial hesitancy could be attributed to procedural complexity and training demands; nonetheless, recently, ESD has been gaining popularity in the USA. This is due to the advancements in endoscopic technology, tailored training programs, and cumulative evidence regarding the efficacy and safety of ESDs. This review aimed to deliberate the historical progress, current implementation, and prospective trajectory of ESDs in the USA. With ongoing clinical research, technological integration, and educational efforts, ESD is likely to become the gold standard for managing large gastrointesitinal lesions. This progress marks an imperative step toward less invasive, more precise, and patient‐centric approaches regarding advanced therapeutic endoscopy in the USA.

## INTRODUCTION

Endoscopic submucosal dissection (ESD), a minimally invasive endoscopic modality, enables the complete resection of superficial gastrointestinal (GI) lesions. Initially developed for treating early gastric cancer, ESD facilitates the en‐bloc resection of complex GI tumors.^1^, ^2^, ^3^, ^4^, ^5^, ^6^ Since its development, the application of ESD has been expanded to include the treatment of esophageal, duodenal, and colorectal lesions, obviating the need for surgical intervention.^6^ ESD exhibits excellent outcomes, including low recurrence rates, microscopically margin‐negative (R0) resections, and improved histopathological assessment, positioning ESD superior to the conventional endoscopic mucosal resection (EMR).^7^

In the Western world, this procedure has initially been met with reservations due to several factors, such as prolonged procedural time, major adverse events, and the requisite expertise to perform the procedure. In the United States of America (USA), cumulative evidence has substantiated the efficacy of ESD; however, the widespread adoption remains delayed. During the past decade, the number of surgical resections for benign colorectal lesions in the USA has increased; moreover, many experienced endoscopists continue to prefer EMR as a treatment modality for both benign and early malignant colorectal lesions.^8^

However, recent advancements in technique and device development have contributed to a heightened understanding of the clinical implications of ESD. Furthermore, the establishment and enhancement of comprehensive training programs are poised to facilitate the eventual adoption of ESD in the USA.

This review aimed to deliberate the historical progress, elucidate the current status, and envisage the prospects of ESD as a minimally invasive procedure in the USA.

### The History of ESD in Japan and the USA

In 1983, Hirao et al. developed a new endoscopic resection technique with local injection of hypertonic saline epinephrine called ERHSE, a pioneering method facilitating the en‐bloc resection of early gastric cancer. This technique encompasses initial marking with a needle knife, using a high‐frequency coagulation current; submucosal lifting of the lesion with a hypertonic mixture solution; circumferential incision using a needle knife; and a snare resection. En bloc resection was achieved in 89% of cases; however, the practicality was hindered by the requirements of two endoscopists and a high skill level.^9^

The limitations of the ERHSE resulted in the development of an insulated‐tip (IT) diathermic knife (IT Knife; Olympus) in 1998.^10^ Characterized by a ceramic ball tip, the IT knife offered a safe peripheral mucosal incision and submucosal dissection, preventing the risk of perforation. Subsequently, various other ESD knives were developed. The HookKnife (Olympus Medical Systems) and FlexKnife (Olympus Medical Systems) were developed in 2001 and 2002, respectively.^1^, ^2^, ^3^, ^4^, ^5^, ^6^, ^7^, ^8^, ^9^, ^10^, ^11^ During this period, the new procedure was referred to by various other names, including “IT‐EMR,” “cutting EMR,” and “exfoliative EMR.” Finally, in 2003, at a consensus meeting in Japan, the term “ESD” was adopted. The Japanese National Health Insurance acknowledged gastric, esophageal, and colorectal ESD in 2006, 2008, and 2012, respectively, thereby initiating widespread acceptance and implementation of ESD in Japan.^12^

In the USA, in 2013, the American Society of Gastrointestinal Endoscopy (ASGE) and the Japanese Gastroenterological Endoscopy Society (JGES) introduced the first society‐endorsed ESD course, the ASGE‐ JGES ESD Course. Thereafter, several educational and continuing medical education ESD courses have been started by industry and academic institutions. In 2021, the ASGE published training guidelines for ESD, which were succeeded by treatment guidelines for upper ESD in 2023.^13^, ^14^

### Factors affecting ESD training

The current ESD training landscape in Japan and East Asia adheres to the traditional apprenticeship model. In their third or fourth year of training, young gastroenterologists engage in a stepwise program that begins with an observation period. During this period, lesion recognition and the procedural environment of ESD are introduced. Subsequently, the trainees undergo hands‐on training, using an animal module to learn the procedural steps. Thereafter, they assist expert endoscopists before being permitted to perform ESD independently under direct supervision.

The initial cases involve small lesions of the distal stomach requiring minimal technical skills. Gradual advancement to more complex cases encompasses the proximal stomach, esophagus, and distal colorectal regions. The training culminates in challenging cases involving the right colon and duodenum, reserved for the final stages due to the technical demands and the need for fine‐skilled craftsmanship.^15^, ^16^

The USA has higher and lower prevalence rates of colorectal and gastric cancer than Asia, respectively. Thus, an increased focus on ESD training is necessary due to the technical difficulty involving colorectal ESDs.^17^ Moreover, variations exist in the exposure to ESD training. ESD training is frequently incorporated into a 1‐year advanced endoscopy fellowship (AEF). Thus, time constraints and the learning curve associated with core advanced endoscopic procedures, including endoscopic ultrasonography (EUS) and endoscopic retrograde cholangiopancreatography (ERCP), limit exposure to ESD training.

**FIGURE 1:**
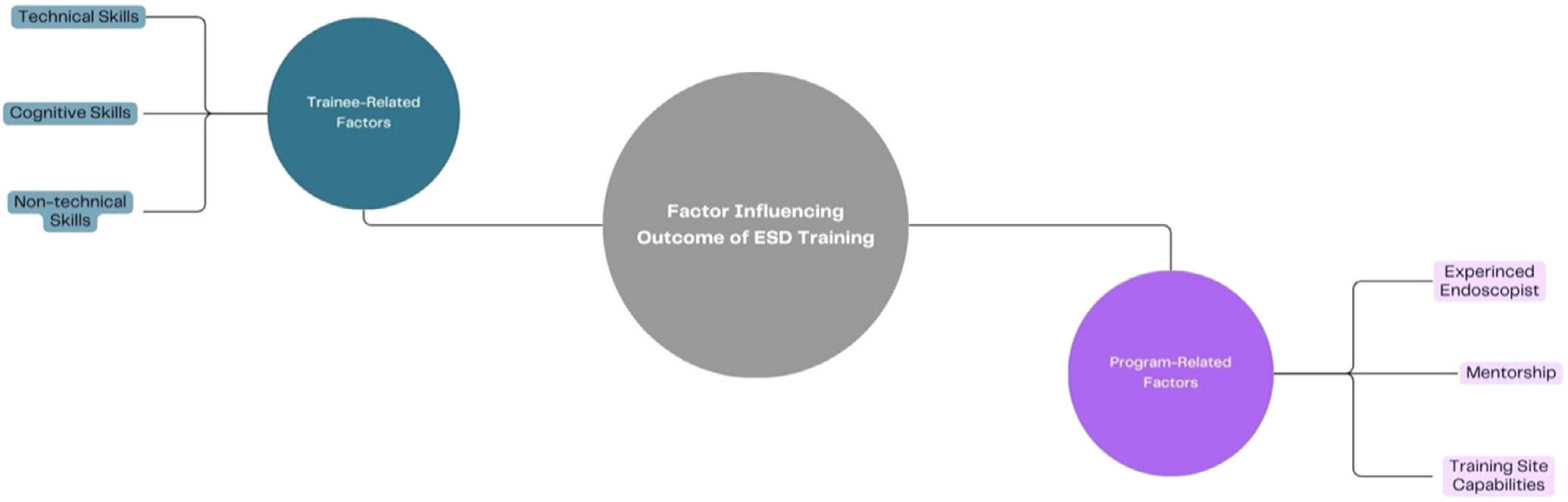
Diagram summarizing the factors influencing the quality of endoscopic submucosal dissection (ESD) training.

The factors influencing the quality of ESD training in the USA can be categorized into two main domains (Figure 1).

#### Trainee‐related factors

##### Technical skills

Proficiency in esophagogastroduodenoscopy (EGD) and colonoscopy is fundamental to the ESD learning curve. One thousand EGDs and 500 colonoscopies are recommended prior to ESD training.^13^, ^18^, ^19^ However, according to our experience, a higher threshold of these procedures may benefit the initial stage of ESD training.^20^ Moreover, the ability to conduct different advanced endoscopic maneuvers, such as EMR, clipping, hemostasis, and endoscopic suturing, should be demonstrated because these serve as rescue techniques in safely and effectively completing ESDs.

The trainee should obtain sufficient experience in ESD using ex vivo models. This exposure aids in acquiring the precise scope maneuvers required for ESD, handling different ESD devices, and developing the technical skills required for proficient ESD performance. A previous study has suggested 30 ex vivo ESD procedures as the threshold for initiating human ESD.^21^

##### Cognitive skills

Cognitive skills, including the ability to assess various GI lesions using the Paris, Japanese Narrow Band Imaging Expert Team, and Kudo classifications, are crucial in enabling the decision‐making capability of endoscopists prior to performing ESD procedures.^22^, ^23^, ^24^

Trainees should discuss the endoscopic findings with ESD experts before each procedure. Additionally, the trainees should understand the strategy for each ESD procedure, including tunneling, pocket, traction techniques, and the procedural steps in ESD in association with gravity.

##### Non‐technical skills

These non‐technical skills include communicating with patients and team members and establishing post‐ESD management plans. Trainees should develop these skills during the training period.

#### Program‐related factors

##### Experienced endoscopist

The training program requires the presence of an experienced endoscopist who is proficient in performing ESDs and is capable of recognizing and facilitating the trainee's learning steps. ESD experts should be familiar with the technical skill benchmarks used in ESDs to assess trainees’ skills periodically.^19^, ^25^

##### Mentorship

Mentorship is a principal feature of a training program and should ideally extend beyond the formal training period. Proctoring support is crucial as trainees transition to independently performing ESDs, particularly during the initial stages after their training.

##### Training site capabilities

The capabilities offered by a training site are pivotal. Adequate equipment, access to ex vivo animal models, and high volumes of referrals for ESDs contribute to the trainees’ exposure to ESDs and improve ESD training outcomes.

### ESD training opportunities in the USA

#### ESD training during the AEF program

Currently, only a few ESD training programs are available in the USA. A primary example is the ESD fellowship program at Brigham and Women's Hospital (BWH). The ESD training is carried out during the 1‐year AEF training program based on a modified master‐apprentice model. The ESD training program is introduced by case observation and assistance. Subsequently, prevalence‐based training is provided, where at the initiation, the trainee is allowed to perform a limited part of the colorectal or esophageal ESD, and the expert endoscopist provides more time as the trainees’ skills improve. This structured 1‐year training module has demonstrated success, with trainees completing 72 ESDs, including 26 independently performed procedures, without assistance under supervision. The adverse event rate was 6.9%, with only one case of microperforation.^20^ The plan for BWH involves a 2‐year training program incorporating EUS, ERCP, bariatric endoscopy, peroral endoscopic myotomy, and ESD, enabling trainees to attain proficiency in various facets of advanced endoscopy.^20^

Furthermore, other institutions offer an additional six months for third‐space endoscopy and ESD training after the 1‐year AFE training program. Currently, some liquidity exists regarding the format of the ESD training programs, resulting from the difficulty of incorporating numerous emerging advanced endoscopic procedures into AEF training.

#### ESD courses

ESD training under the supervision of ESD experts is essential, as it provides direct assessments and immediate feedback in addition to tailored, direct instructions to the trainees. ESD courses are frequently endorsed by societies or sponsored by industries and comprise more than one session. These courses involve expert‐tutored, animal‐model training with varying trainee–trainer ratios and depths of training.

Table 1 summarizes the ESD courses in the USA. ASGE currently offers several courses that provide trainees with official certificates recognizing their knowledge and skills. These courses are called the “Skills, Training, Assessment, and Reinforcement (STAR) Certificate Programs.” These programs include endoscopic suturing, colorectal EMR, and endobariatrics. Nevertheless, ESD is not included in the STAR programs due to the technical complexity and learning curve, requiring multiple attendances. Conversely, certificates of attendance for these courses are provided for hospital credentials.

**TABLE 1:** Endoscopic submucosal dissection (ESD) courses in the United States of America (USA).

Organizer	Course	Hands‐on models	Contents
ASGE	ASGE‐JGES ESD course	Ex‐vivo porcine stomach Live porcine	Didactic lectures Hands‐on training
Olympus America	Introduction to ESD	Ex‐vivo porcine stomach	Didactic lectures Hands‐on training
Olympus America	ESD Master class	Ex‐vivo porcine stomach Live porcine	Didactic lectures Hands‐on training Live case observation
Boston Scientific	Endoluminal surgery ESD course	Ex‐vivo porcine stomach	Online learning Hands‐on training

Abbreviations: ASGE, American Society of Gastrointestinal Endoscopy; ESD, endoscopic submucosal dissection; JGES, Japanese Gastroenterological Endoscopy Society.

#### ESD observership and proctorship programs

Observing ESD procedures performed by experts is known to improve trainees’ ESD performance. In a previous study, a trainee traveled to Japan and observed 43 cases of ESD. He had a significantly decreased post‐observership ESD procedure time than pre‐observership (32.7 ± 15.0 and 61.0 ± 7.4 min, respectively; *p* = 0.0011). Moreover, no perforations were observed post‐observership compared to the 13.8% perforation rate in pre‐observership. Observership programs are currently offered under industry sponsorship (Table 2). Trainees are provided with an opportunity to visit ESD experts’ institutions in the USA to observe the procedures.^25^

**TABLE 2:** Endoscopic submucosal dissection (ESD) observership and proctorship programs.

Organizer	Program
Olympus America	ESD observership and proctorship programs
Boston Scientific	ELSA ESD observership and proctorship programs

Abbreviation: ESD, endoscopic submucosal dissection.

Proctorship, in which ESD experts visit the hospitals where trainees are based, is essential to ensure that trainees safely perform their first few cases after developing their own ESD program. Verbal instructions are provided by the ESD experts regarding the trainees’ procedures and endoscopy room settings, which include the roles the nurses and technicians should play, generator settings, and the selection of devices. Some hospitals in the USA mandate that physicians perform a few proctored ESD procedures prior to performing these procedures independently. These proctorships are supported by several USA companies (Table 2).

### Available ESD devices in the USA

#### ESD knives

**FIGURE 2:**
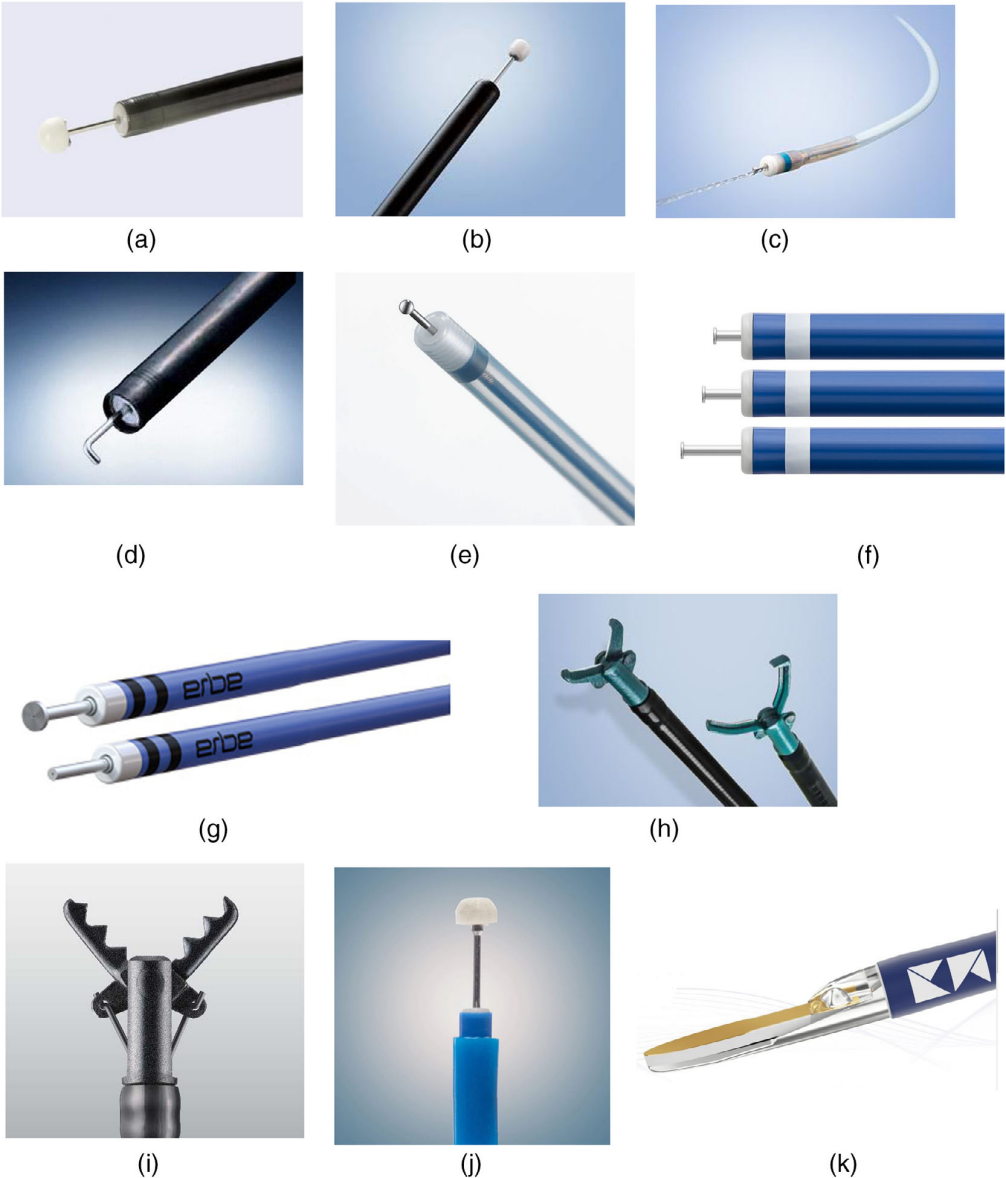
Endoscopic submucosal dissection (ESD) knives available in the United States of America (USA). (a) IT2 knife (Olympus America). (b) IT2 knife nano (Olympus America). (c) DualKnife‐J (Olympus America). (d) HookKnife‐J (Olympus America). (e) Flush Knife (Fujifilm). (f) ORISE ProKnife (Boston Scientific). (g) HybridKnife (ERBE USA). (h) SB knife (Olympus America). (i) Clutch cutter (Fujifilm). (j) ProdGI (Medtronic America). (k) Speedboat (Creo Medical America).

Multiple ESD knives are currently available in the USA. ITKnife2 and ITKnife nano (Olympus America; Figure 2a,b) are classified as IT‐type knives. The DualKnife‐J (Figure 2c), HookKnife‐J (Olympus America; Figure 2d), and Flush Knife (Fujifilm Medical Co.; Figure 2e) offer an integrated through‐catheter injection capability. ORISE ProKnife (Boston Scientific; Figure 2f) and HybridKnife (ERBE USA; Figure 2g) have been equipped with through‐the‐needle injections, allowing for high pressure and better submucosal lifting. The SB knife (Olympus America; Figure 2h) and Clutch‐Cutter knife (Fujifilm; Figure 2i) both provide the advantages of vessel sealing, hemostasis, incision, and dissection. The ProdiGI (Medtronic; Figure 2j) is a multifunctional knife equipped with needle‐type and IT‐type knife functions, whereas the Speedboat (Creo Medical; Figure 2k) has a unique design, utilizing bipolar radio frequency and microwave current.

#### Lifting solutions

**FIGURE 3:**
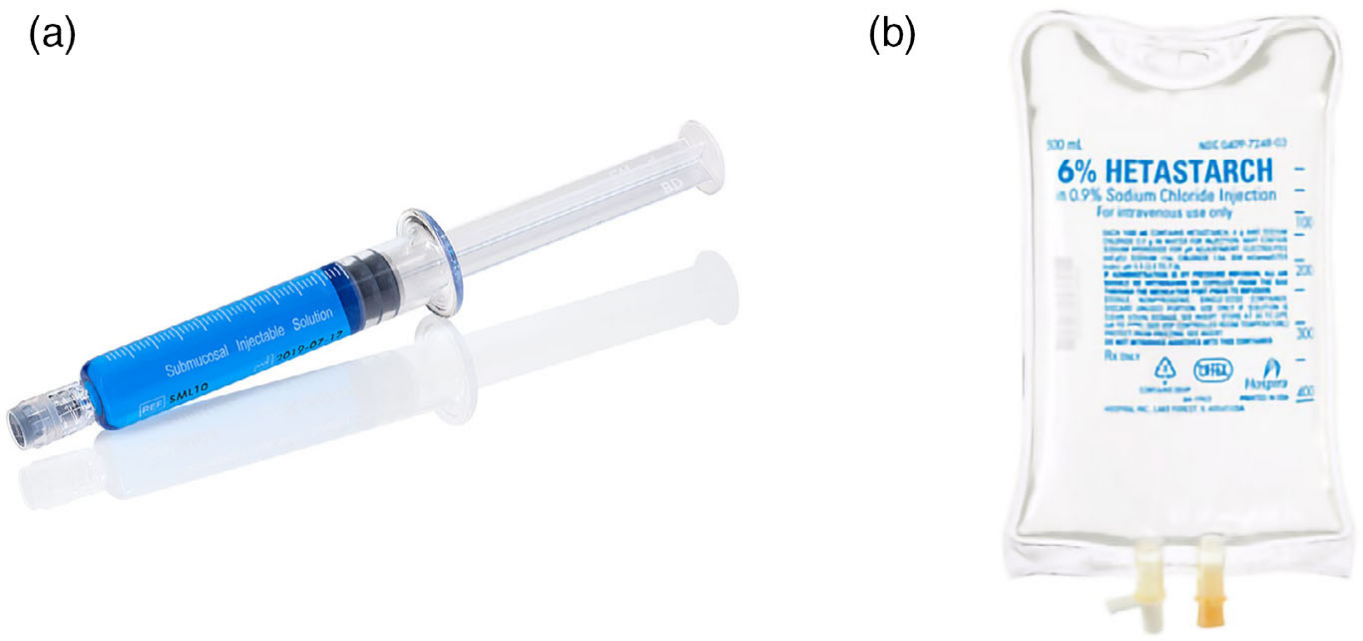
(a) BlueBoost (Micro‐Tech Endoscopy). (b) 6% Hetastarch.

Commercially available lifting solutions in the USA include BlueBoost (Micro‐Tech; Figure 3a), EverLift (Laborie Medical Technologies), Eleview (Medtronic), and Ascendo (STERIS). These solutions are premixed with indigocarmine or methylene blue and sold as a 5‐ or 10‐mL syringe. The 6% hetastarch solution (Figure 3b), a 500‐mL plasma volume expander, is another option as a lifting solution for ESD. Due to the high amount of lifting solution required for ESD, this solution has been used by most ESD practitioners in the USA. Typically, indigocarmine or methylene blue and epinephrine are mixed at the discretion of the endoscopists.

#### Hemostatic devices and agents

**FIGURE 4:**
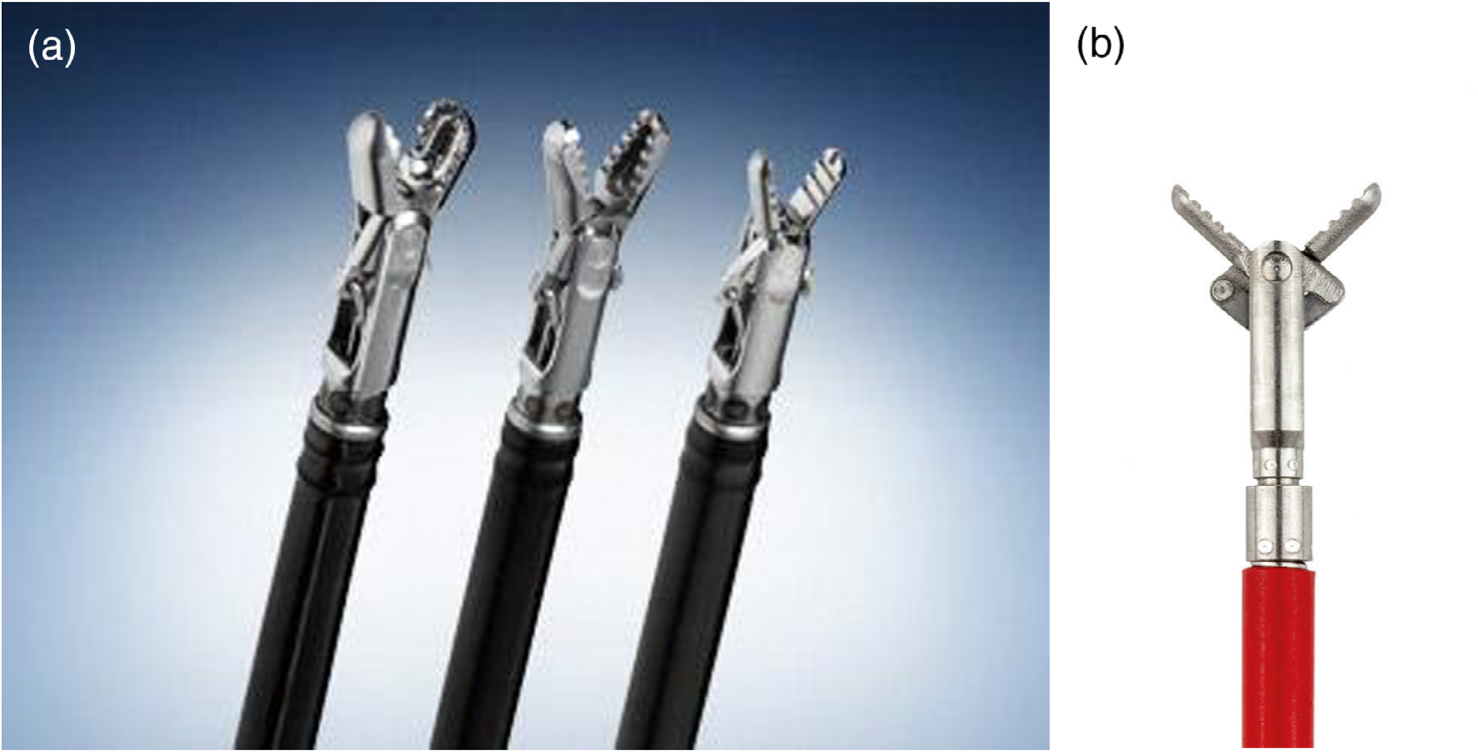
(a) Coagasper (Olympus America). (b) Ensure Grasper (Micro‐Tech Endoscopy).

Bleeding is one of the most commonly encountered adverse events in ESD, with an estimated incidence ranging from 1.8% to 15.6%.^26^, ^27^, ^28^, ^29^, ^30^ To date, various hemostatic devices have been developed, each offering effective hemostasis and optimization of ESD procedures. Monopolar coagulating forceps are available as Coagasper (Olympus America; Figure 4a) and Ensure Grasper (Micro‐Tech Endoscopy; Figure 4b).

The recently developed hemostatic gel, Purastat (3‐D Matrix), is a patented hemostatic absorbable material that provides immediate hemostasis. A previous study has indicated the effectiveness in controlling intraprocedural bleeding;^31^ however, more data are required to confirm this efficacy.

#### Traction devices

**FIGURE 5:**
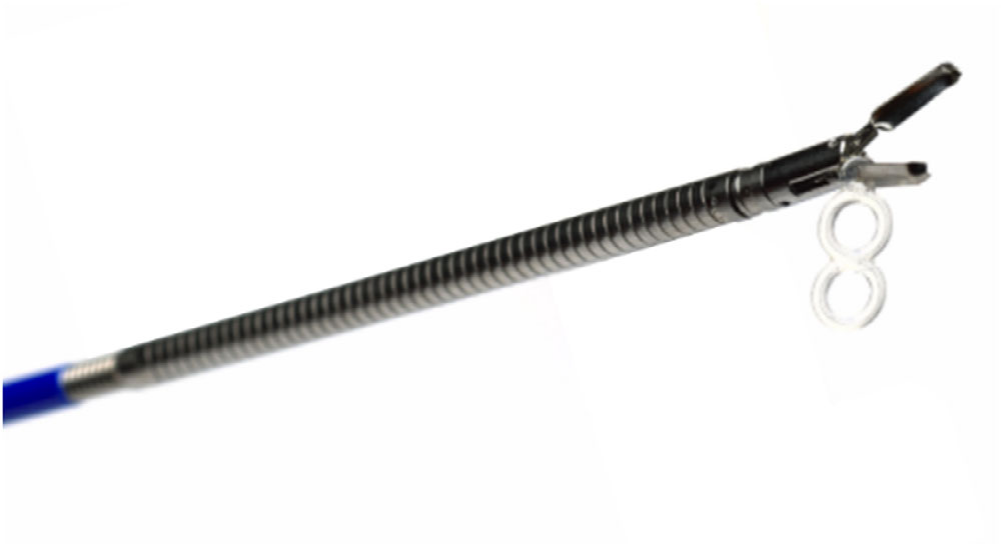
SureTrac (Micro‐Tech Endoscopy).

One of the principal elements for successful ESD is the exposure of the submucosal layer during dissection, which allows for a safer dissection, shorter procedure time, and lower risk of perforation. In Japan, most ESD procedures are performed by utilizing gravity and adjusting the patient's position. However, in the USA, device‐assisted traction is preferred over patient repositioning to shorten the procedure time and improve procedural efficiency. The SureTrac (Micro‐Tech Endoscopy; Figure 5) has the same design as the spring‐and‐loop with clip traction device (S‐O clip).^32^ The SureTrac has recently been approved by the Food and Drug Administration (FDA) and is currently only commercially available, as a through‐the‐scope (TTS) internal traction device. Additionally, the clip‐with‐line method, as described by Oyama et al.,^33^ has been used, particularly for esophageal and proximal gastric ESDs.,^5^ has been used, particularly for esophageal and proximal gastric ESDs.

**FIGURE 6:**
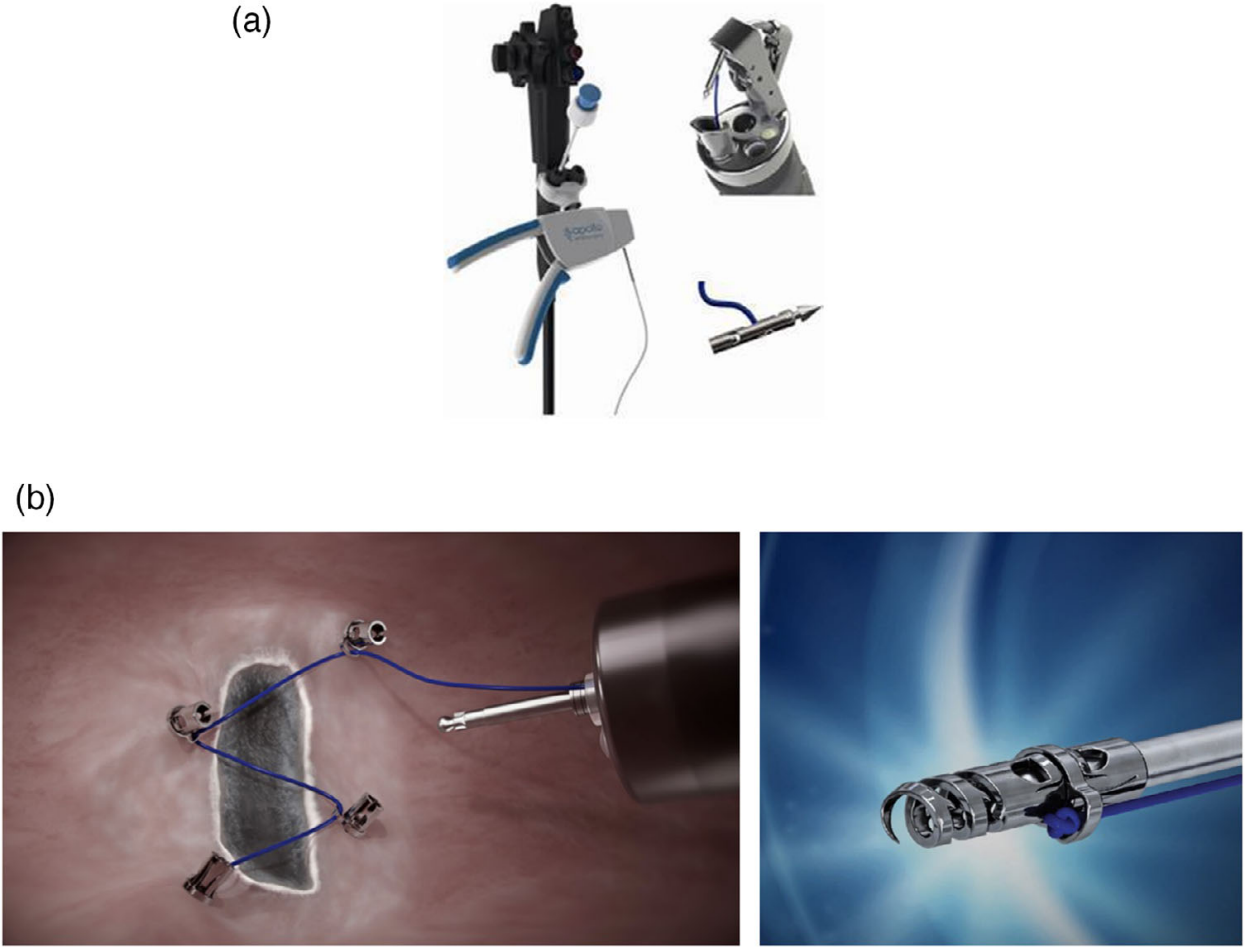
Endoscopic suturing devices: (a) Overstitch device (Apollo Endosurgery/Boston Scientific). (b) X‐Tack device (Apollo Endosurgery/ Boston Scientific).

In 2014, the suture‐pulley method was initially described by Aihara et al.^34^ as a technique that significantly shortens the ESD procedure time when performed by an expert. In this technique, an Overstitch endoscopic suturing device (Apollo Endosurgery/Boston Scientific; Figure 6a) is used to create a suture‐pulley system. After a full‐circumferential mucosal incision, the first stitch is applied to the gastric wall opposite the target lesion. The second stitch is placed at the proximal edge of the mucosal flap, with subsequent deployment of the anchor. The suture is pulled through the esophagus, and tension is applied using the Kelly forceps.

A subsequent, prospective, randomized study has enrolled five attending‐level gastroenterologists and eight GI fellows with no prior experience in ESD. This study has revealed a significant reduction in procedure time and adverse event rates. The advantage of this technique is that an endoscopic suturing device can be used for defect closure. The disadvantage of these techniques is the inability to adjust the direction of tissue retraction.^35^

Traction methods using a sheath, clip/snare, or dedicated device offer excellent adjustability in the traction direction.^36^, ^37^ Multi‐loop traction devices are effective in providing a shorter procedure time for gastric ESD due to the adjustability of the direction.^38^ Adaptive TRACTion is another technique that adds to the safety profile of ESD. Of the 52 patients who underwent ESD using this device, only one and three cases of perforation and delayed bleeding occurred, respectively.^39^ However, these techniques are limited by their inability to reposition the traction points, which are typically required for larger lesions.

**FIGURE 7:**
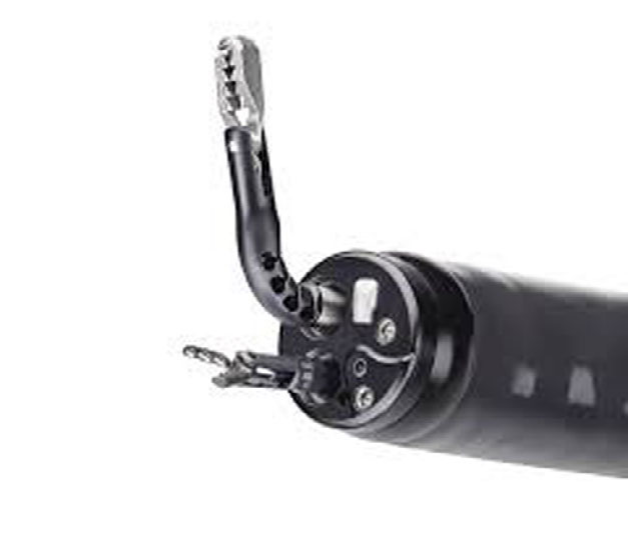
TracMotion (Fujifilm).

In the USA, in 2022, a novel, single‐operator traction device, the TracMotion (Fujifilm Endoscopy; Figure 7), was developed. Compatible with a double‐channel endoscope, this device comprises an articulating grasper and physician control knob, allowing adjustment of the direction of traction and regrasping of the traction points. The TracMotion has been reserved for gastric and rectal ESDs because of the need for a double‐channel endoscope.^40^ Nonetheless, in a pilot, multicenter, ex vivo study that enrolled advanced fellows with limited experience in ESD, this device is effective and less demanding, with a shorter procedure time than that of conventional ESD.^41^

#### Closure devices

Post‐ESD defect closures reduce the risk of bleeding and delayed perforation.^42^, ^43^, ^44^ The Overstitch allows robust and effective closure of both gastric and rectal ESDs, with a high closure rate of 93%–100%.^45^ Moreover, an endoscopic heliX tacking system (X‐Tack device; Apollo Endosurgery/Boston Scientific; Figure 6b) has been developed. This TTS suturing device facilitates defect closure at various locations, including the proximal colon.

**FIGURE 8:**
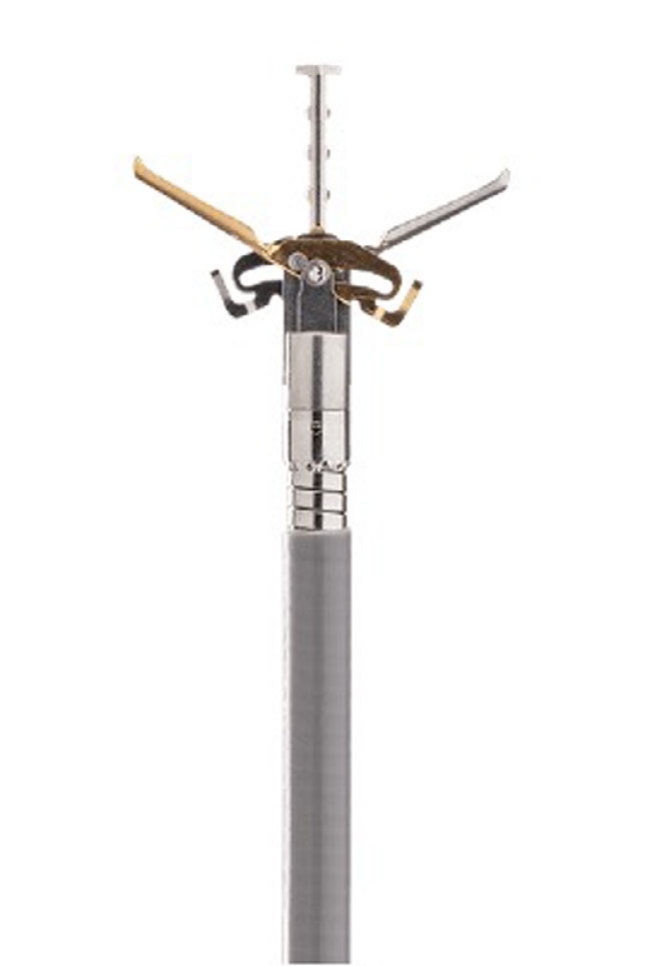
Dual action tissue (DAT) clip (Micro‐Tech Endoscopy).

A previous multicenter USA study has shown that the device is effective and safe, allowing the closure of large defects that are not possible with conventional closure methods.^46^ The device provides a more robust closure with a cost comparable to conventional clip‐closure devices. TTS and over‐the‐scope clips are safe and effective, and only limited experience is required for their application. However, their use has been limited to the closure of small‐to‐medium mucosal defects, resulting in modifications to the technique, such as utilizing a detachable loop or an O‐ring.^47^, ^48^ Recently, a dual‐action tissue clip (Micro‐Tech Endoscopy; Figure 8) has become available in the USA. This clip is equipped with two arms operating independently of each other, with an overall maximum opening width of 15 mm and a 60° angle, which allows approximation of the mucosal edges of large EMR or ESD defects. A recent study by Mohammed et al. has revealed a complete closure rate of 96.3% of EMR or ESD defects with a median size of 40 mm in diameter by using this clip.^49^

### Clinical outcomes of ESD in the USA

Recently, based on the development of new ESD devices and the establishment of effective training systems, the use of ESD has shown rapid growth in the USA. More advanced endoscopists have begun to adopt this technique, resulting in multiple studies showing excellent clinical outcomes of ESD involving various lesion locations (Table 3).

**TABLE 3:** Summary of the endoscopic submucosal dissection (ESD) outcomes in North America.

Study	Lesion	Lesion size	En‐bloc resection rate	R0 resection rate	Curative resection rate	Adverse event rate	Recurrence rate
Draganov et al., 2021^52^	Esophageal 181 (26.2%) Stomach 101 (14.6%) Duodenum 11 (1.5%) Colonic 399 (30.5%) Rectal 188 (27.2%)	40 mm (25–52 mm)	96.7% 98% 91% 85.5% 88.8%	86.2% 82.2% 72.7% 83.4% 85.6%	71.3% 77.2% 72.7% 83.9% 79.8%	Overall 10% Perforation 2.9% Bleeding 2.3%	Overall 5.8% Esophageal 8.9% Stomach 11.1% Duodenum 10% 2.7% colorectal
Ngamruengphong et al., 2021^50^	Stomach 347 (100%)	26.8 ± 16	92.2%	81.8%	‐	Perforation 6.6% Intraprocedural bleeding 1.2% Delayed bleeding 2.6%	Overall 3.9%
Yang et al., 2019^51^	Stomach 171 (100%)	43 mm (34.8–60 mm)	82.5%	74.9%	73.1%	Bleeding 2.3% Perforation 4.1%	Overall 0.6%
Ge et al., 2019^53^	Right colon 44 (53%) Left colon 11 (13.3%) Rectum 28 (33.7%)	47.5% ± 27.7 mm	97.4%	97.4%	93.5%	Perforation 1.3% Bleeding 3.9%	None

A study regarding gastric ESD by Ngamruengphong et al. has demonstrated en bloc and R0 resection rates of 92.0 and 82.0%, respectively. Moreover, perforation, delayed bleeding, and recurrence rates were 6.6%, 2.6%, and 3.9%, respectively.^50^ A study that included 171 patients by Yang et al. regarding rectal ESD has revealed en bloc and R0 resection rates of 82.5% and 74.9 %, respectively.^51^

Recently, a multicenter North American study^52^ has revealed that among the 692 patients with esophageal, gastric, duodenal, and rectal lesions, with a median lesion size of 40 mm, ESD encompassed en bloc and R0 resection rates of 91.5% and 84.2%, respectively. These high success rates were accompanied by lower rates of adverse events, primarily with bleeding at 2.9% and only 20 (2.3%) cases of perforation. These adverse events were amenable to endoscopic management, with only one case requiring surgery.

A recent study by Ge et al.^53^ has highlighted the current issues in referral patterns for ESD in the USA. In a study population of 77 patients, tattoos that had been previously applied affected the dissection process and were encountered in 16.9% (*n* = 13) of cases. Moreover, prior EMR attempts were noted in 29.9% (*n* = 23) of cases. Mild and severe fibrosis were observed in 31.2% (*n* = 24) and 23.4% (*n* = 18) of cases, respectively. Regarding the multivariate analysis, the presence of tattoos predicted the failure to achieve curative resection (odds ratio [OR] = 0.13; 95% confidence interval [CI] = 0.02–0.98; *p* = 0.048). A lesion size > 50 mm (OR = 3.89; 95% CI = 1.13–13.41; *p* = 0.031), presence of tattoo (OR = 9.38; 95% CI = 1.05–83.83; *p* = 0.045), and prior EMR attempts (OR = 7.13; 95% CI = 1.76–28.90; *p* = 0.006) predicted a procedure time of ≥90 min. Thus, awareness of these factors among referring physicians is crucial. EMR attempts or the application of tattoos near the lesion site should be avoided because the outcomes of ESDs may be negatively affected.

### Future perspectives

Thus far, all ESD procedures have been performed using conventional single‐channel flexible endoscopes. Understanding the challenging nature of ESD and the limitations of traditional endoscopy has fueled the development of robotic endoscopic (RE) technology.^54^, ^55^ RE potentially provides an alternative approach for performing ESD with greater precision, safety, and effectiveness. The significant advantages of RE include the ability to handle tissue bimanually, facilitating effective traction and countertraction, and allowing triangulation, resulting in a wider working space for both robotic arms. These aspects of the RE systems enable faster, safer, and more effective intraluminal tissue resection.

**FIGURE 9:**
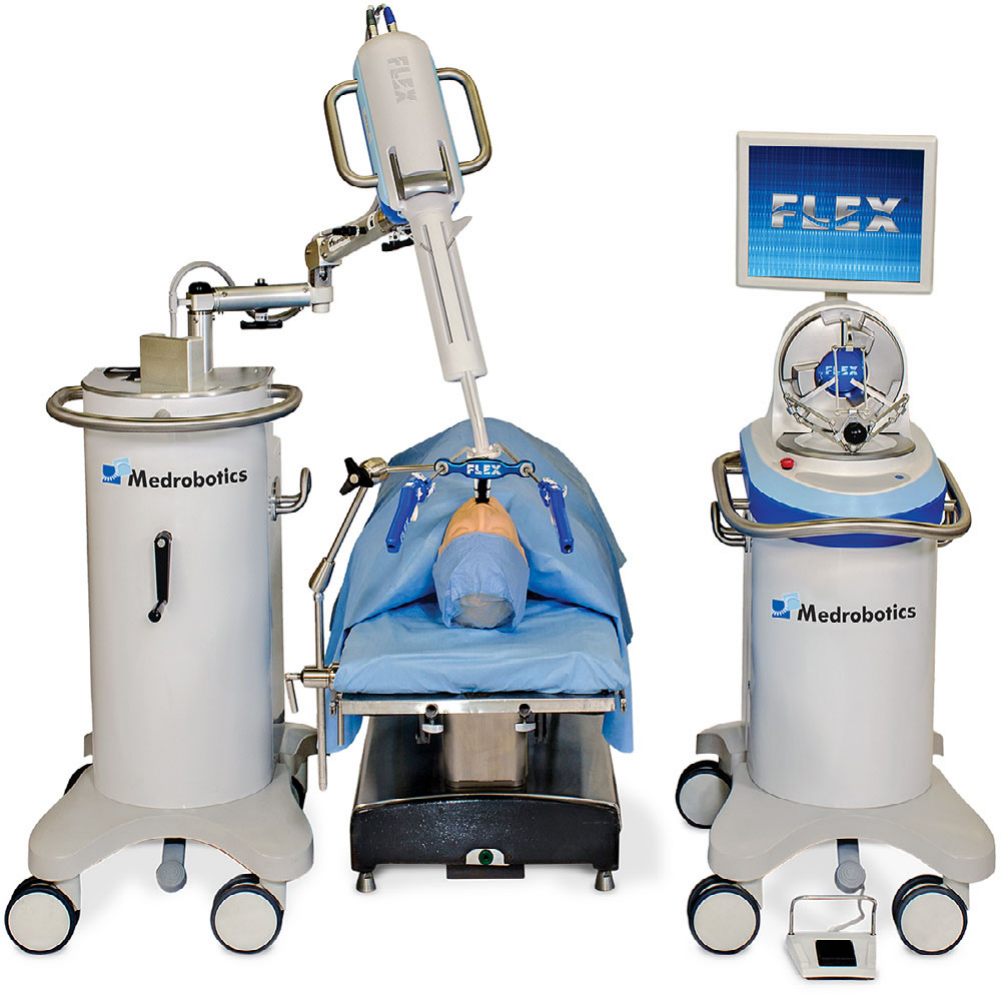
Flex robotic system (Medrobotics).

Initially approved by the FDA in 2017, the first RE system developed in the USA is the Flex Robotic System (Medrobotics; Figure 9).^56^, ^57^ In a proof‐of‐concept, ex‐vivo, randomized, controlled pilot study, we enrolled endoscopists with no experience in robotic‐assisted ESD (RESD) and conventional ESD.^58^ RESD has revealed a superior outcome with a 100% completion rate compared to the 50% completion rate of conventional ESD (*p* < 0.0001). Moreover, RESD resulted in a significantly shorter procedure time than conventional ESD (34.1 min ± 19.14 and 88.6 min ± 31.40, respectively; *p* = 0.001).

**FIGURE 10:**
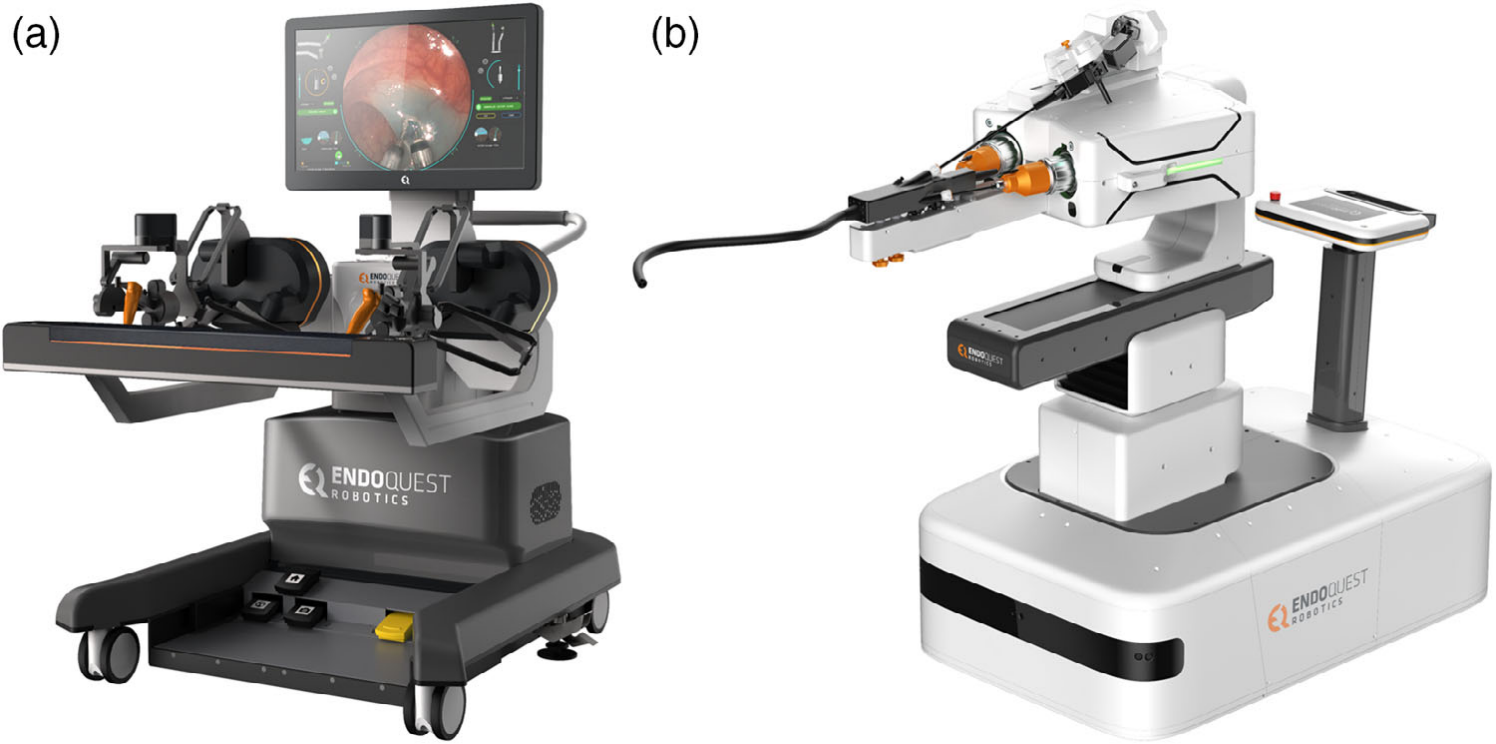
Flexible endoscopic robotic system (EndoQuest Robotics): (a) Physician console. (b) Patient cart.

Recently, another flexible endoscopic robotic system has been developed, the Endoquest robotic system (EndoQuest Robotics; Figure 10a,b). This is the first fully robotic endoluminal platform that incorporates a robotic overtube, endoscope, and a range of exchangeable and flexible instruments, all controlled via a single physician console. We have conducted a prospective, randomized study by enrolling 10 GI fellows with no prior experience in RESD and conventional ESD, similar to our previous study. This study has revealed a significantly higher en bloc resection rate, shorter procedure time, and reduced risk of perforation.

Despite the excellent outcomes of these preclinical studies, studies involving human participants are required to elucidate procedural outcomes. Moreover, the learning curve requires further clarification regarding the handling of the robotic device for both endoscopists and surgeons. However, with continued advancements in robotic technologies, the potential for expanding the application exists.

## SYNOPSIS

ESD represents a paradigm shift as a minimally invasive procedure for managing superficial GI lesions, offering a compelling alternative to traditional endoscopic and surgical approaches. Since its inception, ESD has undergone considerable advancements, with extended applicability throughout the GI tract and improved en‐bloc and curative resection rates. ESD has been robustly adopted in Japan and other East Asian countries. However, this procedure has encountered a cautious reception in the Western hemisphere, largely due to procedural complexity, demanding technical proficiency, and prolonged learning curves.

Nonetheless, ESD has gradually been embraced in the USA, propelled by the cumulative evidence of the efficacy, refinements of the technique, and the emergence of comprehensive and tailored training programs. These programs aim to bridge the proficiency gap by enhancing gastroenterologists’ cognitive and technical skills. The evolving ESD landscape of the USA is reflected in the increasing number of training programs, the development of state‐of‐the‐art ESD devices, and the formulation of guidelines that integrate ESD into the clinical armamentarium for GI lesions.

Robotic endoscopic technology has the potential to overcome the traditional ESD's limitations. As this technology matures and is integrated into ESD, a more streamlined procedure, mitigated associated risks, and decreased skill level are anticipated. Thus, enhanced patient outcomes and a broadened scope for clinical utility will ensue.

## CONCLUSION

ESD is poised for broader acceptance in the USA. With ongoing clinical research, technological integration, and educational efforts, ESD will likely become the gold standard for managing large GI lesions. This progress marks an imperative step toward less invasive, more precise, and patient‐centric approaches regarding advanced therapeutic endoscopy.

## CONFLICT OF INTEREST STATEMENT

Abdulrahman Qatomah has nothing to declare. Hiroyuki Aihara is a consultant for Olympus America, Fujifilm Medical Systems, Boston Scientific, ConMed, MicroTech, BioDevek, and Endoquest Robotics. Hiroyuki Aihara receives a research fund from Boston Scientific.
